# Investigation of the Prevalence of *Toxoplasma gondii* in Meat, Meat Organs, Milk, Dairy Products and Eggs in Different Animals, in Iran

**DOI:** 10.1002/vms3.70654

**Published:** 2025-10-21

**Authors:** Amirhossein Nasiri, Ayub Ebadi Fathabad, Fatemeh Salmani, Asma Afshari, Tayebeh Zeinali

**Affiliations:** ^1^ Student Research Committee Birjand University of Medical Sciences Birjand Iran; ^2^ Department of Nutrition and Food Hygiene School of Health, Social Determinants of Health Research Center Birjand University of Medical Sciences Birjand Iran; ^3^ Department of Epidemiology and Biostatistics School of Health Geriatric Health Research Center Birjand University of Medical Sciences Birjand Iran; ^4^ Department of Nutrition School of Medicine Mashhad University of Medical Sciences Mashhad Iran; ^5^ Department of Nutrition and Food Hygiene School of Health Geriatric Health Research Center Birjand University of Medical Sciences Birjand Iran

**Keywords:** egg, food, meat, milk, *Toxoplasma gondii*

## Abstract

*Toxoplasma gondii* is an obligate intracellular protozoan parasite which final host is the cat and causes infection in humans and domestic animals. The main source of infection is the consumption of contaminated food such as meat, milk and their products. The aim of this study was to investigate the prevalence of *T. gondii* in meat, meat organs, milk, dairy products and eggs of different animals in Iran. This study was conducted by searching electronic databases such as Magiran, Scientific Information Database (SID), Ganj, PubMed and Scopus from 2000 to 2025. The findings of this study showed that in molecular analysis, the highest contamination of meat and meat organs was in sheep meat (78%) in Isfahan, and the lowest contamination was in beef meat (0%) in Semnan. In milk and dairy products, the highest contamination was found in goat milk (20%) in East Azerbaijan. The highest contamination of eggs was also found in Astara, Kermanshah and Jahrom (12.2%). In conclusion, red meat and milk had the highest and lowest contamination among investigated foods. As all types of food had contamination with this parasite, they must be consumed thoroughly cooked.

## Graphical abstract

The prevalence of *Toxoplasma gondii* in the food samples was 0.17. The highest and lowest prevalence of *T. gondii* in food was seen in meat and milk and dairy products as 0.22 and 0.06, respectively. Forest plot of prevalence of *Toxoplasma gondii* in Iran.

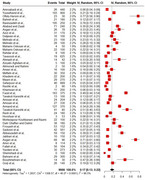

## 1 Characteristics of *Toxoplasma gondii*

*Toxoplasma gondii* infection is a common parasitic disease worldwide. This parasite is of the order Apicomplexa, subclass Coccidia, class Aspirozoacida, family Sarcocystidae, genus *Toxoplasma* and species *T. gondii*. The final host of this parasite is the cat, which causes severe infections in pets and humans (Lima and Lodoen 2019). *T. gondii* infects warm‐blooded animals, including humans, following contact with felines (especially cats) as a dedicated host (Amroabadi et al. 2021). *T. gondii* is one of the most important parasites shared by humans and animals. Almost all warm‐blooded animals, including marine mammals and marsupials, are infected, but cats, especially feral cats, are the only known definitive hosts of the *T. gondii* parasite. Pregnant women and people with weakened immune systems have more severe complications from the disease caused by the *T. gondii* parasite. The most important symptoms of this disease are microcephaly, intracranial calcifications and retinochoroiditis. Other symptoms of this disease include hearing loss, blindness, mental disorders, epilepsy, anaemia, jaundice and encephalitis (Parmley et al. 1992). There are three main subtypes of *T. gondii*, which do not differ significantly clinically but can be distinguished for epidemiological investigations: tachyzoites (as a complex), bradyzoites (in tissue cysts) and sporozoites (in oocysts) (Basirpour 2022).

*T. gondii* has two stages of sexual and asexual reproduction. In the definitive host, which is the cat, both sexual and asexual cycles occur, but in other hosts, or intermediate hosts, only the asexual phase occurs. Intermediate hosts such as humans are infected by the ingestion of tissue cysts containing bradyzoites and haemocysts containing sporozoites. In the absence of appropriate medical care or in the presence of a suppressed immune system, the patient may succumb to the death of the patient. However, following the administration of the drug and the development of the immune system, a resistant membrane is formed around the parasite within the nucleated cells, resulting in the formation of a tissue cyst. Upon maturation, the host's nucleated cells and the tissue cyst are released into the tissue, subsequently spreading to different organs (Wendte et al. 2011).

The infection with *T. gondii* is transmitted by ingestion of food contaminated with sporulated oocysts and consumption of contaminated milk, raw or undercooked meat contaminated with tissue cysts. If milk or tissues infected with the parasite are consumed by humans, that person is capable of transmitting the disease for the rest of their lives, because the cysts remain in the host's body for the rest of its life. Transmission can occur vertically and iatrogenically through organ transplantation and faecal‐oral (Jones and Dubey 2012; Djurković‐Djaković et al. 2019; Sadeghi et al. 2022). In Iran, a number of studies have been conducted in various fields, including pathogenesis, epidemiology, vaccine preparation, treatment and others (Rostami et al. 2018; Sadeghi et al. 2022). However, a review study to collect and provide a summary of data on the prevalence of *T. gondii* in some foods such as meat, milk and eggs has not been done yet. This study aimed to investigate the prevalence of *T*. gondii in meat, milk, its products and eggs in various animals in Iran.

### 1.1 Toxoplasmosis

Toxoplasmosis is a disease caused by the parasite *T. gondii* and is known to be a common disease between humans and animals (Djurković‐Djaković et al. 2019). Toxoplasmosis is the third disease in the world that causes people to be hospitalized due to foodborne infection. In Iran, the average prevalence of toxoplasmosis in the human population is reported to be 39.3% (Basirpour 2022). The virulence of *T. gondii* is such that it usually parasitizes the host without causing clinical disease. It only occasionally causes severe clinical manifestations. *T. gondii* may initially disseminate to the mesenteric lymph nodes and subsequently to distant organs by invading the blood and lymph nodes, replicating in almost every cell of the body. All extracellular forms of the parasite are directly affected by antibody, whereas intracellular forms are not (Fentress and Sibley 2011). *T. gondii* infects humans by ingestion of undercooked meat or products containing tissue cysts or consumption of water and food contaminated with mature oocysts. It is noteworthy that direct contact with cats is not a prerequisite for the transmission of *T. gondii*, as the oocysts can remain viable in the environment for an extended period. It has been observed that the excretion of faeces and oocysts from cats may be higher in hot and humid areas, which may indicate a higher prevalence of disease in tropical regions (Basirpour 2022). Domestic animals infected with the *T. gondii* parasite can contaminate meat, which represents an important source of infection for humans (Kalantari et al. 2014).

Anaemic patients and pregnant women are groups that consume raw meat and its products. They believe in the health benefits of these products (Djurković‐Djaković et al. 2019). Toxoplasmosis in a healthy person is usually asymptomatic and improves spontaneously. However, in pregnant mothers, this condition causes foetal toxoplasmosis, and its diagnosis is difficult. The age of the foetus at the time of infection is the most significant factor affecting the future of the foetus (Dunn et al. 1999). The risk of foetal infection in the first 13 weeks of the mother's pregnancy is approximately 15%, while the risk of infection in the 36th week is approximately 72%. There is an inverse relationship between foetal age and the severity complications caused by congenital toxoplasmosis. It can be observed that the severity of the disease in a baby who is infected in the first trimester of pregnancy is greater than in the second trimester and any other time (Kieffer and Wallon 2013). The risk of clinical manifestations such as organ failure and foetal death in utero and severe neurological complications such as microcephaly, hydrocephaly, retinochoroiditis and mental retardation in the first 3 months of pregnancy is higher than any other time. If the infection occurs in the third trimester of pregnancy, the baby is usually asymptomatic at birth (Dunn et al. 1999; Kalantari et al. 2014). Since *T. gondii* is of great importance as a foodborne agent, and given that milk, meat and eggs are among the most important foods worldwide, further research and studies on it will be important and practical. Therefore, this study aimed to investigate and compare the molecular prevalence of *T. gondii* in samples of milk, meat, their products and eggs from different animals.

The prevalence of toxoplasmosis in sheep has been reported to range from 28.5% to 78% worldwide. Additionally, the prevalence of this disease in sheep and goats in different regions is estimated to be between 24.5% and 33.3% (Basirpour 2022). Diagnosis of *T. gondii* is typically conducted using serological tests, molecular‐based methods and fluorescent microscopy. Serological tests are highly sensitive, although in some cases, the results may show false negatives. Additionally, the fluorescent microscope is accurate but requires live cysts. Finally, molecular‐based techniques are considered the most specific and sensitive methods and were chosen as suitable and accurate methods for diagnosing this disease (Kalantari et al. 2014; Liu et al. 2015). One of the most common serological diagnostic methods of *T. gondii* is Enzyme Linked Immunosorbent Assay (ELISA) and OdiT ELISA, as well as the most common molecular diagnostic method of this parasite is polymerase chain reaction (PCR) methods, especially nested_PCR (Bahadory et al. 2013; Amroabadi et al. 2021).

## 2 Methods

### 2.1 Data Sources and Search Strategy

The study was conducted by searching in electronic databases, including the Magiran, Scientific Information Database (SID), Ganj, PubMed and Scopus, from 2000 to 2025. Furthermore, a manual search was conducted on Google Scholar and theses. The following keywords were employed in the search: The search terms included ‘*T. gondii*’ AND ‘Iran’, AND ‘molecular’, AND ‘Food’ OR ‘milk’, OR ‘meat’, OR ‘milk products’, OR ‘meat products’ OR ‘Egg’.

### 2.2 Inclusion Criteria

The present study includes cross‐sectional studies on the molecular prevalence of *Toxoplasma* in milk, dairy products, meat, their edible products and eggs in Iran between 2000 and 2025. Also, in this review, original articles were selected in which the PCR method was used.

### 2.3 Exclusion Criteria

In the present study, the exclusion criteria include the following: 1_review articles, 2_articles from the years before 2000 to investigate the prevalence of *T. gondii* in food, 3_data from serological methods (due to not being related to the main topic of our article), 4_molecular data related to the prevalence of *T. gondii* in non‐edible organs or aborted foetuses and 5_articles that did not report the exact sample size and number/percentage of *T. gondii*.

### 2.4 Quality Control

The Joanna Briggs Institute was used to assess and review the quality of the studies (Munn et al. 2014). Articles that scored 6 out of 10 were considered suitable for extraction. Duplicate articles were also removed with the help of endnote reference manager.

### 2.5 Data Extraction

Data extraction was performed using Microsoft Excel. Finally, according to the Joanna Briggs checklist, articles that received a quality score of more than 60% were used in this review. The following information from the studies was extracted: First author, publication date, study design, study location, number of samples, sample source (type of animal, including cattle, goat, sheep, camel, buffalo and hen/chicken), sample type (red meat, white meat, milk and dairy products and egg) and diagnostic methods (Nested_PCR, PCR and restriction fragment length polymorphism [RFLP]_PCR).

### 2.6 Statistical Analysis

The data were analysed by use of R software (version 4.2) with metafor packages. In this study, meta‐analysis was used to systematically gather and analyse effect sizes from various research findings, allowing for a comprehensive synthesis of existing data. *I* ^2^ statistic was used to report heterogeneity among studies.

## 3 Results and Discussion

**FIGURE 1:**
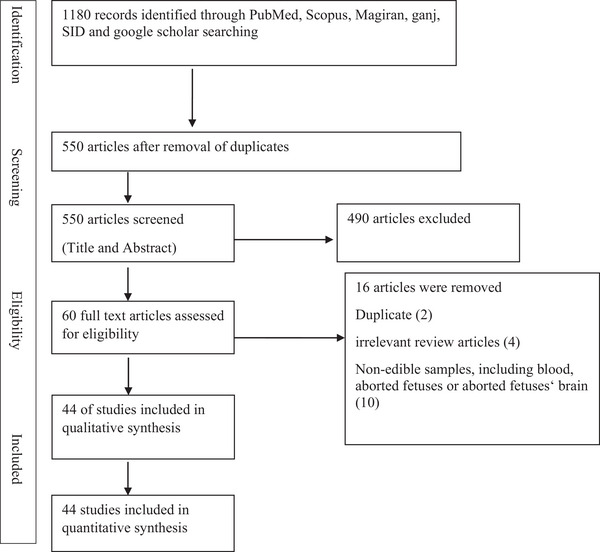
Diagram of identification and selection of studies for inclusion in the review.

A total of 1180 articles were identified for this study. Among them, 44 studies were selected for data extraction. Figure 1 shows the PRISMA guideline for reporting the articles.

### 3.1 Detection Methods

It is imperative to employ robust and effective diagnostic techniques to ascertain the risk of toxoplasmosis. In this review, we will examine some of the molecular and serological tests that have been developed. The latex agglutination test (LAT), the indirect haemagglutination antibody test (IHA), the modified agglutination test (MAT), the indirect fluorescent antibody test (IFAT) and ELISA were significant serological tests employed in the studies.

ELISA test: ELISA stands for enzyme‐linked immunosorbent assay. This method is one of the most specific and highly sensitive methods for determining the presence of antibodies in toxoplasmosis (Balsari et al. 1980). The ELISA system comprises a solid antibody or antigen, an enzyme‐labelled antigen or antibody and a substrate for reaction with the enzyme. This method is employed for the detection of both antibodies and antigens. One of the most significant ELISA methods for the diagnosis of *T. gondii* is indirect ELISA. Indirect ELISA is employed to ascertain antibody titre in serum samples or to identify specific antibodies. The fundamental principle underlying the test is that diluted serum is typically added to coated antigens in the solid‐phase microwell or well. Following the addition of the sample and the completion of the incubation period and washing step, the antihuman globin labelled with the enzyme is added to the well. The ELISA test is increasingly prevalent in the detection of antigens (infectious agents) and antibodies due to its simplicity and sensitivity. However, one of the limitations of the ELISA method is its transient readings and restricted antigenic information (Liu et al. 2015).

LAT: In this test, animal or human antigens are employed. This reaction occurs when the body recognizes a pathogen and creates a specific antibody for a specific antigen (protein structure present on the surface of the pathogen). The LAT test has a lower entrance fee than ELISA, is relatively stable to perform, and the interpretation of the results is faster and easier. LAT test is a simple, fast, inexpensive and relatively stable test that does not cross‐react with other antibodies. It has been largely replaced other serological tests. One of the limitations of the LAT test is that the pH, osmolarity and ion concentration of the solution influence the extent of binding the conditions under which the LAT is performed (Kumar et al. 2019).

The indirect fluorescent test (IFA): It is a safe diagnostic method that does not use live tachyzoites. It is based on the antibody reaction with specific antigen isolated from killed *Toxoplasma* tachyzoites. The result of this reaction is detected by adding fluorescent anti‐IgM or IgG to the patient's serum and using a fluorescence microscope. The effective application of this method necessitates consideration several factors, including the nature of the antigen, the specificity and sensitivity of the primary antibody, the properties of the fluorescent label, the permeabilization and sample fixation method and the fluorescence imaging of the cell (Liu et al. 2015).

MAT: The MAT test is a serological method for the detection of *T. gondii*, which is relatively simple and cost‐effective compared to the ELISA test. Additionally, there is no species restriction (Mainar‐Jaime and Barberán 2007).

IHA: The antigen employed in the indirect haemagglutination test for the detection of antibodies against *T. gondii* is derived from this parasite via a specific methodology. These antigens contain special polysaccharides that have different active serological components. Serologically active components are capable of detecting antibodies produced against cell wall antigens, which are predominantly IgM. This method is advantageous in that it does not necessitate the presence of multiple witnesses, thereby reducing the likelihood of error and accelerating the testing process (Salek Moghaddam et al. 2001).

A number of molecular tests are employed to identify the organism, including PCR, real‐time PCR test (qPCR), loop‐mediated isothermal amplification (LAMP), PCR‐RFLP and nested method of PCR (Nested_PCR) (Gharekhani et al. 2018; Yousefvand et al. 2021).

PCR: Conventional diagnostic methods are limited by, such as light microscopy, the possibility of errors in the detection of morphological forms and the inability to identify certain species. To overcome these limitations, different PCR tests have been developed to identify *T. gondii* by targeting specific genes, including the repetitive gene 1B, the rDNA, the 30P gene, and the ITC_1 (internal transcribe spacer). PCR is a useful diagnostic tool for the acute stage of the disease. This method is a very sensitive method for detecting cases where *Toxoplasma* parasites are visible in the blood. In contrast, the ELISA method based on Tox_IgG can detect certain *Toxoplasma* antibodies in the chronic stage of the disease, but it is less accurate in confirming acute toxoplasmosis. Among the disadvantages of this method, we can mention cross‐reaction with various factors, as well as the lack of proper efficiency in people with immune deficiencies (Liu et al. 2015).

The qPCR test is a method that determines the quantity of PCR products with the help of fluorescent technology. A qPCR test utilizing a FRET protocol targeting a repetitive gene region of 529 bp was employed for the detection of *T. gondii* in immunocompromised patients and pregnant women. This was compared to a qPCR test based on a TaqMan protocol targeting the SRNA18 gene and to a nested PCR which targets *T. gondii* B1 gene. The results demonstrated that qPCR test is a more specific and sensitive method. Furthermore, qPCR is a qualitative or semi‐quantitative test. Consequently, it is not feasible to quantify the expression of a specific gene with the real‐time method (Lin et al. 2000).

LAMP: LAMP is a method based on DNA amplification that has been shown to be a valuable tool for the rapid diagnosis of *T. gondii*. This method is based on the detection of a 200–300 repeat sequence of 529 bp fragments in *T. gondii*. The method of performing the LAMP test involves maintaining a constant temperature of 64°C throughout the reaction, 1 h (with the loop primer within 35 min). This method is a rapid and reliable diagnostic tool in the acute stage of toxoplasmosis, particularly in developed countries. Furthermore, the lamp is highly specific, initiating the reaction with four primers that are capable of covering six regions of the target gene sequence (Ameri et al. 2019).

PCR‐RFLP test: RFLP is a method that employs the examination of alterations in homologous DNA sequences to distinguish population's species or to identify gene locations in a sequence. In RFLP analysis, a DNA sample is fragmented into smaller units by one or more restriction enzymes, and the resulting restriction fragments are separated by size using gel electrophoresis. The polymorphism method is relatively straightforward and inexpensive, and the obtained pattern is repeatable and reliable. The RFLP method necessitates a series of complex steps and typically requires several weeks to yield the desired results. In contrast, techniques such as PCR can rapidly amplify and determine target DNA sequences within hours. Additionally, the RFLP method is relatively expensive (Fatahi et al. 2007; Iraji et al. 2016).

Nested_PCR test: Nested PCR is a method that enhances the sensitivity and specificity of the PCR reaction. This reaction involves the use of two sets of external and internal primers against a target sequence in two consecutive PCR reactions. In fact, nested PCR consists of two consecutive PCR reactions in which the external primer amplifies a piece of DNA in the first round according to the target sequence of the primers. In the second round, the PCR reaction product of the first round is used as a template, and the internal primers do not bind to any dimer primers or non‐specific products produced in the first round of PCR by binding to the template. Consequently, the technique of nested PCR is advantageous in maintaining the specificity of PCR through a multitude of combined primary and secondary PCR cycles. Another advantage of the nested PCR technique is the replication of small amounts of DNA and the reduction of target sequence‐specific replication. However, the limitations and problems of the nested PCR technique include the high probability of contamination and the high cost of this technique (Fallahi et al. 2014).

Diagnosis based on serological tests and detection of anti‐*T. gondii* antibodies is also one of the other protozoan diagnostic methods. The antibody test is useful for screening a large number of samples in a short time. One of the disadvantages of diagnosis based on serology is the possibility of cross‐reactions and the high margin of error of this method. The advantages of PCR‐based methods are high speed and accuracy, high sensitivity and specificity, and safety. One of the disadvantages of the conventional PCR method is that it may require more steps for final diagnosis. Among the methods based on PCR, the nested_PCR method provides very high characteristics for gene amplification due to the use of more primers (Meftahi et al. 2021; Raieszadeh et al. 2021).

Finally, it was found that in terms of performance and application, the most common method for serological investigations was ELISA (antibody). Similarly, for the molecular investigation of *T. gondii* in meat, milk, dairy products and eggs, the most sensitive and widely used method is Nested_PCR.

### 3.2 Prevalence of *T. gondii*

**FIGURE 2:**
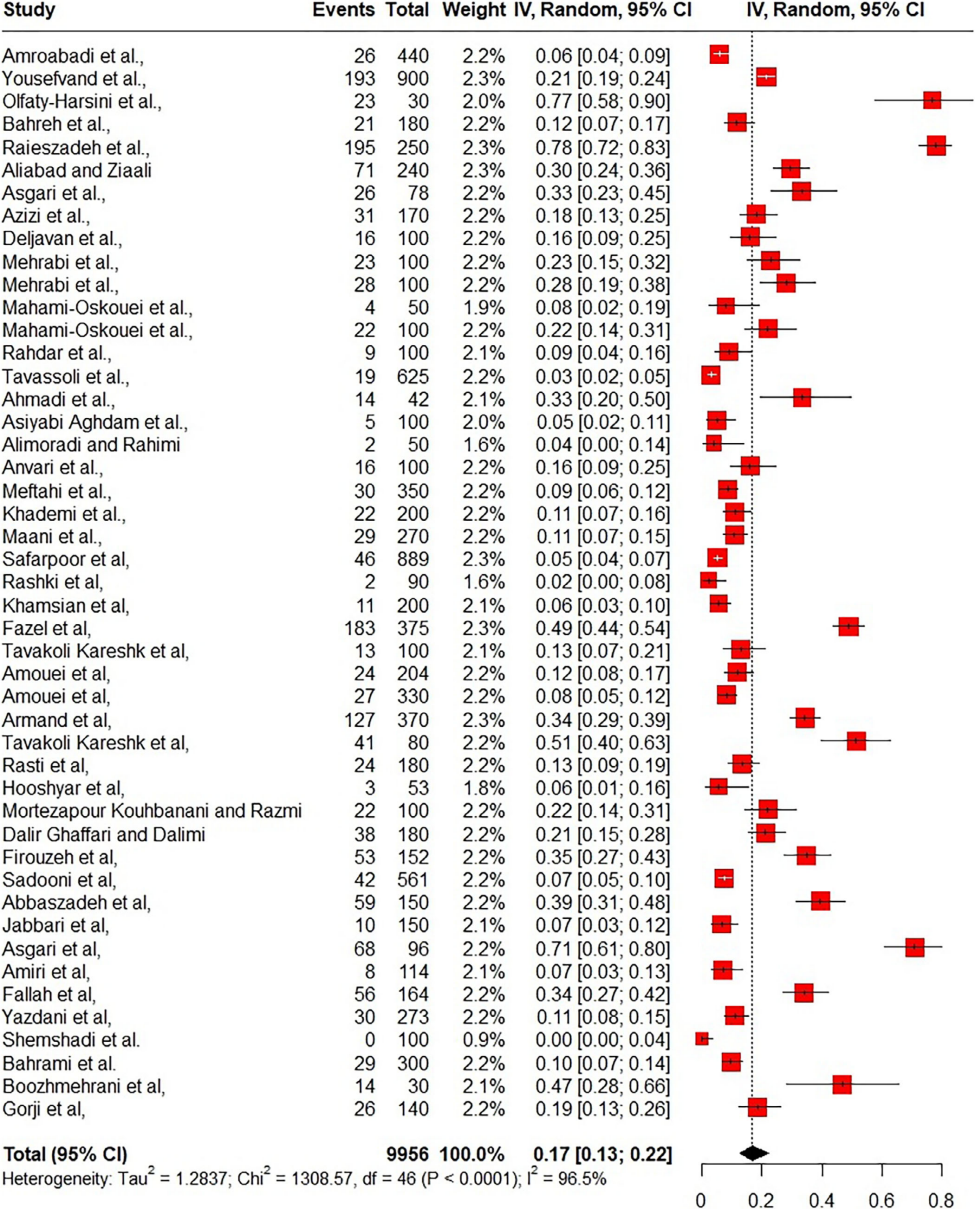
Forest plot of prevalence of *Toxoplasma gondii* in Iran.

A series of searches were conducted on meat, milk, dairy products and eggs, which yielded disparate results. The findings revealed a significant discrepancy in the prevalence of *T. gondii* infection across different geographical regions of Iran (Sadeghi et al. 2022). In general, in this review study, it was determined that the prevalence of *T. gondii* in the samples was 0.17 (95% CI: 0.13–0.22; *I* ^2^: 96.5%) (Figure 2).

Molecular analysis showed that the highest prevalence of *T. gondii* in meat and its products was observed in sheep meat in Isfahan province and the lowest prevalence in cattle in Ahvaz (Rahdar et al. 2012; Raieszadeh et al. 2021). Similarly, the highest prevalence of *T. gondii* in milk and its products was observed in goat milk in East Azerbaijan province and the lowest prevalence in buffalo milk in East Azerbaijan (Deljavan et al. 2022; Asiyabi Aghdam et al. 2023). The details of the investigated studies are presented in Table 1.

**TABLE 1:** Characteristics of studies investigated *Toxoplasma gondii* in foods.

Food	Detection method	Prevalence (sample number)	Location	Reference
Sheep milk	Nested_polymerase chain reaction (PCR)	8% (100)	Isfahan, Chaharmahal and Bakhtiari, Khuzestan	Amroabadi et al. (2021)
Goat milk	Nested_PCR	7.5% (80)	Isfahan, Chaharmahal and Bakhtiari, Khuzestan	
Camel milk	Nested_PCR	4/44% (90)	Isfahan, Chaharmahal and Bakhtiari, Khuzestan	
Cattle milk	Nested_PCR	5% (100)	Isfahan, Chaharmahal and Bakhtiari, Khuzestan	
Buffalo milk	Nested_PCR	4.28% (70)	Isfahan, Chaharmahal and Bakhtiari, Khuzestan	
Sheep milk	PCR	6.48% (185)	Tehran, Isfahan, Fars	Safarpoor et al. (2013)
Goat milk	PCR	9.44% (180)	Tehran, Isfahan, Fars	
Camel milk	PCR	2.5% (160)	Tehran, Isfahan, Fars	
Buffalo milk	PCR	3.65% (164)	Tehran, Isfahan, Fars	
Cattle milk	PCR	3.5% (200)	Tehran, Isfahan, Fars	
Sheep milk	PCR	2.2% (90)	Sistan	Rashki et al. (2024)
Goat milk	Nested_PCR	5.5% (200)	Yazd	Khamsian et al. (2021)
Sheep meat	PCR	22% (150)	Ahvaz	Yousefvand et al. (2021)
Sheep heart	PCR	32% (150)	Ahvaz	
Sheep liver	PCR	17.3% (150)	Ahvaz	
Goat meat	PCR	17.3% (150)	Ahvaz	
Goat heart	PCR	24% (150)	Ahvaz	
Goat liver	PCR	16% (150)	Ahvaz	
Sheep meat	Nested‐PCR	26.67% (30)	Khorramabad	Olfaty‐Harsini et al. (2017)
Brain	Nested‐PCR	50% (30)	Khorramabad	
Boar (tongue, muscle, diaphragm and heart)	PCR	46.7% (30)	Shush, Shushtar, Dezful and Abadan	Boozhmehrani et al. (2025)
Cattle (Tissue, heart and diaphragm)	PCR	56% (125)	Jahrom	Fazel et al. (2021)
Camel (heart and diaphragm)	Nested_PCR	26% (50)	Kerman, Razavi Khorasan, and South Khorasan	Tavakoli Kareshk et al. (2018)
Sheep brain	PCR	18.57% (140)	Semnan	Gorji et al. (2018)
Sheep heart	PCR	12.58% (151)	Mazandaran	Amouei et al. (2022)
Goat heart	PCR	9.4% (53)	Mazandaran	
Chicken heart	PCR	9.46% (243)	Mazandaran	
Duck heart	PCR	4.6% (87)	Mazandaran	
Sheep (heart and diaphragm)	PCR	34.32% (370)	Jahrom	Armand et al. (2016)
Sheep (heart, brain, and diaphragm)	PCR	56.66% (40)	Kerman, Razavi Khorasan, and South Khorasan	Tavakoli Kareshk et al. (2017)
Goat (heart, brain, and diaphragm)	PCR	44.16% (40)	Kerman, Razavi Khorasan, and South Khorasan	
Sheep meat	Nested_PCR	14.4% (90)	Yazd	Bahreh et al. (2021)
Goat meat	Nested_PCR	8.8% (90)	Yazd	
Sheep heart	Nested_ PCR	78% (250)	Isfahan	Raieszadeh et al. (2021)
Cattle meat	PCR	0% (100)	Semnan	Shemshadi et al. (2018)
Sheep meat	PCR	17.8% (90)	Kashan	Rasti et al. (2018)
Goat meat	PCR	8.9% (90)	Kashan	
Cattle meat	PCR	5.7% (53)	Kashan	Hooshyar et al. (2021)
Sheep diaphragm	PCR	37.5% (40)	Sabzevar	Aliabadi and Ziaali (2016)
Sheep heart	PCR	22.5% (40)	Sabzevar	
Goat diaphragm	PCR	35% (40)	Sabzevar	
Goat heart	PCR	17.5% (40)	Sabzevar	
Camel diaphragm	PCR	45% (40)	Sabzevar	
Camel heart	PCR	20% (40)	Sabzevar	
Sheep heart	Nested_PCR	22% (100)	Mashhad	Mortezapour Kouhbanani and Razmi (2020)
Cattle (diaphragm and heart)	Nested_PCR	21.1% (180)	Tehran	Dalir Ghaffari and Dalimi (2019)
Sheep diaphragm	Nested_PCR	47.8% (46)	Quchan	Firouzeh et al. (2023)
Sheep heart	Nested_PCR	26.1% (46)	Quchan	
Goat heart	Nested_PCR	23.3% (30)	Quchan	
Goat diaphragm	Nested_PCR	40% (30)	Quchan	
Goat (heart, diaphragm and tongue)	Nested_PCR	18.2% (187)	Jahrom	Sadooni et al. (2022)
Chicken heart	Nwsted_PCR	39.3% (150)	Guilan	Abbaszadeh et al. (2022)
Sheep (brain, tongue, liver, and muscles of neck, intercostals and femoral)	PCR	37.5% (56)	Shiraz	Asgari et al. (2011)
Goat (brain, tongue, liver, and muscles of neck, intercostals and femoral)	PCR	22.7% (22)	Shiraz	
Sheep liver	PCR	8% (150)	Ahvaz	Bahrami et al. (2019)
Goat liver	PCR	11.3% (150)	Ahvaz	
Sheep (red meat, liver, tongue and brain)	Nested_PCR	38% (50)	Chaharmahal va Bakhtiari	Azizi et al. (2014)
Cattle (red meat, liver, tongue and brain)	Nested_PCR	8.57% (70)	Chaharmahal va Bakhtiari	
Sausage	Nested_PCR	12% (50)	Chaharmahal va Bakhtiari	
Sheep milk	PCR	11.11% (45)	East Azerbaijan	Deljavan et al. (2022)
Goat milk	PCR	20% (45)	East Azerbaijan	
Donkey milk	PCR	20% (10)	East Azerbaijan	
Chicken	PCR	23% (100)	Semnan	Mehrabi et al. (2023)
Egg	PCR	28% (100)	Semnan	
Sheep meat	RFLP_ PCR	28% (50)	East Azerbaijan	Mahami‐Oskouei et al. (2017)
Cattle meat	RFLP_ PCR	16% (50)	East Azerbaijan	
Chicken meat	RFLP PCR	8% (50)	East Azerbaijan	
Cattle meat	PCR	9%(100)	Tabriz	Jabbari et al. (2024)
Buffalo meat	PCR	2% (50)	Tabriz	
Sheep meat	PCR	14% (50)	Ahvaz	Rahdar et al. (2012)
Cattle meat	PCR	4% (50)	Ahvaz	
Sheep milk	RFLP_PCR	4.63% (345)	Urmia	Tavassoli et al. (2013)
Goat milk	RFLP_PCR	1.07% (280)	Urmia	
Chicken brain	PCR	38.1% (21)	Khoramabad	Ahmadi et al. (2020)
Chicken meat	PCR	28.57% (21)	Khoramabad	
Chicken meat	Nested_PCR	70.83% (96)	Shiraz	Asgari et al. (2009)
Chicken meat	PCR	7.06% (114)	Kashan	Amiri et al. (2023)
Meat products (salami, sausage, hamburger and kebab)	PCR	34% (164)	East Azerbaijan	Fallah et al. (2011)
Meat products (salami, sausage, hamburger, hams and frankfurters)	PCR	10.98% (273)	Chaharmahal va Bakhtiari	Yazdani et al. (2014)
Camel milk	PCR	13.33% (15)	East Azarbaijan	Asiyabi Aghdam et al. (2023)
Cattle meat	PCR	3.63% (55)	East Azarbaijan	
Buffalo milk	PCR	3.33% (30)	East Azarbaijan	
Cattle cheese (dairy products)	Nested_PCR	6.7% (30)	Kashan	Alimoradi and Rahimi (2022)
Cattle cream (dairy products)	Nested_PCR	0% (10)	Kashan	
Cattle butter (dairy products)	Nested_PCR	0% (10)	Kashan	
Cattle meat	PCR	16% (100)	Sistan and Baluchestan	Anvari et al. (2018)
Cattle milk	PCR	5.33% (150)	Alborz	Meftahi et al. (2021)
Sheep milk	PCR	12% (100)	Alborz	
Goat milk	PCR	10% (100)	Alborz	
Egg	RFLP_PCR	11% (200)	Bandar Abbas	Khademi et al. (2018)
Egg	PCR	12.2% (90)	Astara, Kermanshah, Jahrom	Maani et al. (2023)
Quail egg	PCR	4.4% (90)	Astara, Kermanshah, Jahrom	
Duck egg	PCR	15.5% (90)	Astara, Kermanshah, Jahrom	

### 3.3 Prevalence of *Toxoplasma gondii* in Various Foods

According to the results obtained from this study, the highest prevalence of *T. gondii* in food was seen in red and white meat as 0.22 (95% CI: 0.15–0.30; *I* ^2^: 96.2%) and 0.22 (95% CI: 0.1–0.43; *I* ^2^: 96.2%), respectively, and the lowest prevalence was in milk and dairy products as 0.06 (95% CI: 0.04–0.08; *I* ^2^: 75.9%). The prevalence of *T. gondii* in eggs was 0.15 (95% CI: 0.08–0.28; *I* ^2^: 89.4%) (Supporting Information section).

### 3.4 Prevalence of *Toxoplasma gondii* in Food Animals

In food animals, the highest prevalence rate of *T. gondii* with a value of 0.21 (95% CI: 0.14–0.30; *I* ^2^: 95.4%) was in sheep and chicken 0.21 (95% CI: 0.10–0.38; *I* ^2^: 94.1%). The lowest prevalence rate with a value of 0.04 (95% CI: 0.02–0.06; *I* ^2^: 0%) was in Buffalo. The prevalence of this parasite in cattle was 0.07 (95% CI: 0.04–0.11; *I* ^2^: 63.8%), in camel 0.10 (95% CI: 0.04–0.24; *I* ^2^: 89.8%), and in goat 0.13 (95% CI: 0.09–0.19; *I* ^2^: 85.6%) (Supporting Information section).

### 3.5 Meat and Meat Products

In the molecular testing of sheep meat and meat products, the highest prevalence was 78% in Isfahan (Raieszadeh et al. 2021) and the lowest was 8% in Ahvaz (Bahrami et al. 2019). In cattle, the highest reported prevalence was 56% (cattle liver) in Jahrom, and the lowest was 3.63% in East Azarbaijan (Fazel et al. 2021; Asiyabi Aghdam et al. 2023) and 0% in Semnan (Shemshadi et al. 2018). In goat meat, the highest reported prevalence was 44.16% (goat heart, brain and diaphragm) in Kerman, Razavi Khorasan and South Khorasan, and the lowest reported prevalence was 8.8% in Yazd (Tavakoli Kareshk et al. 2017; Bahreh et al. 2021). Almost 45% of contamination was observed in camel meat in Sabzevar (Aliabadi and Ziaali 2016). In Shiraz province, the prevalence of *T. gondii* in chicken was reported as 70.83% (Asgari et al. 2009). In general, according to this study, sheep meat and meat products were more contaminated than others, which may be due to the climate, cultural and ethnic diversity, and the way animals interact with cats in these cities (Isfahan). In general, raw or undercooked meat from cattle, camels, sheep and goats is a potential source of *T. gondii* and should not be consumed by at‐risk groups of the population (Belluco et al. 2016).

### 3.6 Milk and Dairy Products

Molecular tests performed on sheep's milk and dairy products showed the highest prevalence with 12% in Alborz province and the lowest prevalence with 2.2% in Sistan (Meftahi et al. 2021; Rashki et al. 2024). In cattle milk, the highest prevalence of 5.33% was reported in Alborz province, and the lowest prevalence of 0% (cattle butter and cream) was reported in Tehran, Isfahan and Fars provinces (Meftahi et al. 2021; Alimoradi and Rahimi 2022). In goat milk, the highest reported prevalence was 20% in East Azerbaijan province, and the lowest prevalence was 1.07% in Urmia (Tavassoli et al. 2013; Deljavan et al. 2022). In camel milk, the highest reported contamination was 13.33% in East Azerbaijan province, and the lowest prevalence was 2.5% in Tehran, Isfahan and Fars provinces. *T. gondii* infection has also been reported in buffalo milk, with the highest prevalence of 4.28% in Isfahan, Chaharmahal va Bakhtiari and Khuzestan provinces, and the lowest prevalence of 3.33% in East Azerbaijan province (Amroabadi et al. 2021; Asiyabi Aghdam et al. 2023). Overall, goat milk samples had higher contamination (in East Azerbaijan), which may be due to the type of climate in this region (semi‐arid), the type of livestock farming and the high contact of livestock and other animals with cats. Contamination of raw milk and dairy products with *T. gondii* can occur through ways such as cross‐contamination and lack of hygiene during milking, storage and transportation (Amroabadi et al. 2021). Optimal vaccination of dairy herds, especially goat herds, against the parasite *T. gondii* can prevent the transmission of this protozoan to raw milk (Meftahi et al. 2021).

### 3.7 Egg

Molecular tests on eggs showed that the highest prevalence of this parasite in eggs (hen) was in Semnan with 28%, and the lowest prevalence in quail eggs as 4.4% was in Astara, Kermanshah and Jahrom (Maani et al. 2023; Mehrabi et al. 2023). *T. gondii* infection is more important in free‐range native poultry than in industrial poultry and poultry farms (Ahmadi et al. 2020). According to surveys, the highest amount of egg (hen) contamination was observed in Semnan. This outbreak may be due to the type of housing, climate, poor hygiene and more contact between eggs and cats in these areas.

## 4 Conclusion

*T. gondii* parasite is a food‐born parasite with high epidemiological relevance, distributed in different cities of Iran, and due to its complex life cycle, its identification and diagnosis are difficult. In the molecular survey, the most contaminated food was meat, and the least was milk. Regarding food animals, sheep and chicken had the highest and buffalo the lowest contamination with the parasite. Considering that the *T. gondii* parasite is a threat to human health, more caution is needed when using meat, meat products or eggs in immunocompromised consumers and pregnant women. Therefore, controlling the entry of felines, especially cats, into domestic animal facilities is essential to prevent primary and secondary contamination of milk, meat, eggs and their products. There were less studies on the prevalence of this parasite in dairy products, poultry eggs and animals like camel and buffalo that should be investigated in future studies.

## Author Contributions

**Amirhossein Nasiri**: investigation, writing – original draft, methodology, validation, writing – review and editing. **Ayub Ebadi Fathabad**: investigation, writing – original draft, writing – review and editing, methodology. **Fatemeh Salmani**: writing – original draft, writing – review and editing, formal analysis, data curation. **Asma Afshari**: writing – original draft, writing – review and editing, methodology. **Tayebeh Zeinali**: conceptualization, investigation, writing – original draft, writing – review and editing, validation, methodology, project administration, supervision.

## Ethics Statement

This research was performed according to international code of ethics.

## Conflicts of Interest

The authors declare no conflicts of interest.

## Supporting information




**Supplementary File 1**: vms370654‐sup‐0001‐SuppMat.docx

## Data Availability

Data presented in the article.
