# Supplementary figures and images for: Investigation of the Prevalence of Toxoplasma gondii in Meat, Meat Organs, Milk, Dairy Products and Eggs in Different Animals, in Iran
Source: Vet Med Sci. 2025 Oct 21;11(6):e70654. doi: 10.1002/vms3.70654 (PMC12538642; doi:10.1002/vms3.70654)

Buffalo

Chicken

Cattle

Camel


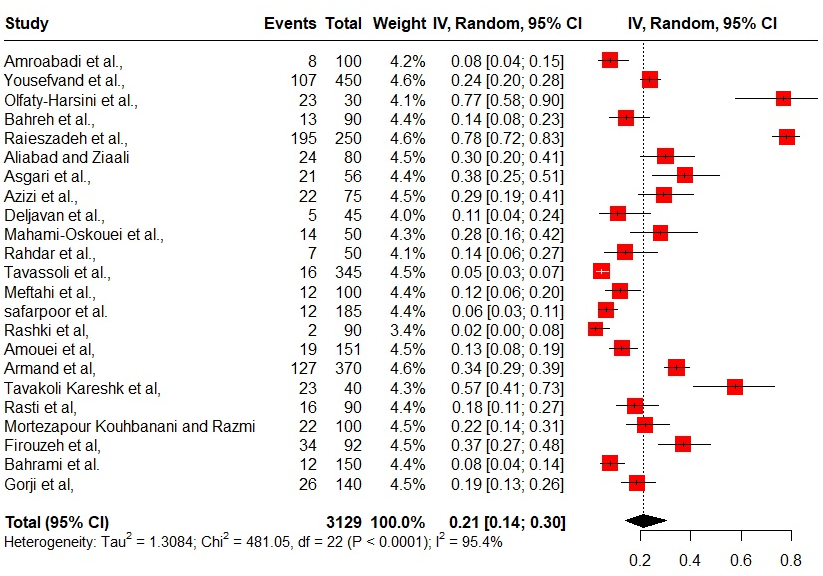


Sheep


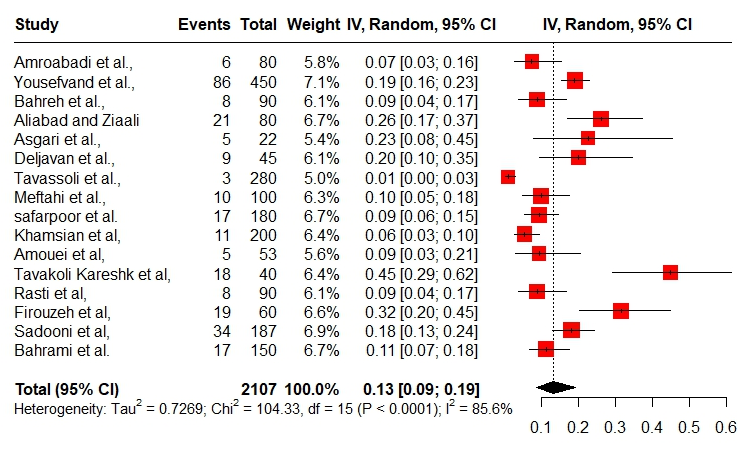


Goat

Milk


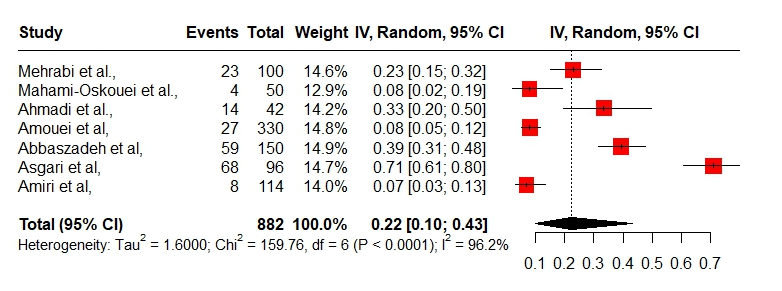


White meat

Red meat

Egg

Supplement: Supplementary file 1 — vms370654‐sup‐0001‐SuppMat.docx [file VMS3-11-e70654-s001.docx]
